# Role of zinc and **α**_2_macroglobulin on thymic endocrine activity and on peripheral immune efficiency (natural killer activity and interleukin 2) in cervical carcinoma

**DOI:** 10.1038/sj.bjc.6690040

**Published:** 1999-01

**Authors:** E Mocchegiani, A Ciavattini, L Santarelli, A Tibaldi, M Muzzioli, P Bonazzi, R Giacconi, N Fabris, G G Garzetti

**Affiliations:** Immunology Centre, Research Department, Institute National Research Centres on Aging (INRCA), Ancona, Italy; Institue of Gynaecology and Obstetrics, University of Ancona, Ancona, Italy

**Keywords:** zinc, α_2_-macroglobulin, thymulin, interleukin 2, soluble interleukin 2 receptor, natural killer activity, cancer

## Abstract

Decreased natural killer (NK) activity as well as interleukin 2 (IL-2) are risk factors for the progression of cervical carcinoma. NK activity and IL-2 may be thymus controlled. Plasma levels of active thymulin, a zinc-dependent thymic hormone (ZnFTS), are reduced in cancer because of the low peripheral zinc bioavailability. Zinc and thymulin are relevant for normal immune functions. α2-Macroglobulin is an inhibitor of matrix metalloproteases (MMPs) against invasive tumour proliferation. Because α_2_-macroglobulin has a binding affinity (*K*_d_) for zinc that is higher than does thymulin, it may play a key role in immune efficiency in cancer. Plasma samples of 22 patients (age range 35–60 years) with locally advanced squamous cervical carcinoma and with FIGO stage Ib2–IIb were examined. They showed reduced active thymulin, decreased NK activity and IL-2 production, increased soluble IL-2 receptor (sIL-2R) and augmented α_2_-macroglobulin in the circulation, whereas plasma zinc levels were within the normal range for age. Significant positive correlations were found between zinc or active thymulin and α_2_-macroglobulin (*r* = 0.75, *P*< 0.01, *r* = 0.78, *P*< 0.01, respectively) in cancer patients. In vitro zinc increases IL-2 production from peripheral blood mononuclear cells (PBMCs) of cancer patients. These data suggest that an increase in α_2_-macroglobulin, which competes with thymulin for zinc binding, may be involved in causing a thymulin deficit with a consequent decrease of IL-2 and NK cytotoxicity. Thus, physiological zinc treatment in cervical carcinoma maybe restores impaired central and peripheral immune efficiency. © 1999 Cancer Research Campaign


British Journal of Cancer (1999) 79(2), 244–250
© 1999 Cancer Research Campaign

### Role of zinc and $\alpha_2$ macroglobulin on thymic endocrine activity and on peripheral immune efficiency (natural killer activity and interleukin 2) in cervical carcinoma

**E Mocchegiani[1], A Ciavattini[2], L Santarelli[1], A Tibaldi[1], M Muzzioli[1], P Bonazzi[2], R Giacconi[1], N Fabris[1] and GG Garzetti[2]**

[1]Immunology Centre, Research Department, Institute National Research Centres on Aging (INRCA), Ancona, Italy; [2]Institue of Gynaecology and Obstetrics, University of Ancona, Ancona, Italy

**Summary** Decreased natural killer (NK) activity as well as interleukin 2 (IL-2) are risk factors for the progression of cervical carcinoma. NK activity and IL-2 may be thymus controlled. Plasma levels of active thymulin, a zinc-dependent thymic hormone (ZnFTS), are reduced in cancer because of the low peripheral zinc bioavailability. Zinc and thymulin are relevant for normal immune functions. $\alpha$2-Macroglobulin is an inhibitor of matrix metalloproteases (MMPs) against invasive tumour proliferation. Because $\alpha_2$-macroglobulin has a binding affinity ($K_d$) for zinc that is higher than does thymulin, it may play a key role in immune efficiency in cancer. Plasma samples of 22 patients (age range 35–60 years) with locally advanced squamous cervical carcinoma and with FIGO stage Ib2–IIb were examined. They showed reduced active thymulin, decreased NK activity and IL-2 production, increased soluble IL-2 receptor (sIL-2R) and augmented $\alpha_2$-macroglobulin in the circulation, whereas plasma zinc levels were within the normal range for age. Significant positive correlations were found between zinc or active thymulin and $\alpha_2$-macroglobulin ($r = 0.75$, $P < 0.01$, $r = 0.78$, $P < 0.01$, respectively) in cancer patients. In vitro zinc increases IL-2 production from peripheral blood mononuclear cells (PBMCs) of cancer patients. These data suggest that an increase in $\alpha_2$-macroglobulin, which competes with thymulin for zinc binding, may be involved in causing a thymulin deficit with a consequent decrease of IL-2 and NK cytotoxicity. Thus, physiological zinc treatment in cervical carcinoma maybe restores impaired central and peripheral immune efficiency.

**Keywords:** zinc; $\alpha_2$-macroglobulin; thymulin; interleukin 2; soluble interleukin 2 receptor; natural killer activity; cancer

Cell-mediated immunity and natural killer (NK) activity play an important role in immunosurveillance in neoplasia (Burnet, 1971; Broder and Negson, 1982). Recent studies suggested interesting relationships between decreased natural cytotoxicity and tumour spread (Robertson and Ritz, 1990). NK activity is significantly decreased in advanced cervical carcinoma compared with early stage (Garzetti et al, 1996) and associated with an impairment of interleukin 2 (IL-2) production (Rani et al, 1992), representing a risk factor for the progression of neoplasia (Shiffman and Brinton, 1995). IL-2 is required for NK activity (Henny et al, 1981), and both may be also thymus controlled (Goldstein, 1984). Some models of cancer (Mocchegiani et al, 1990, 1994) show reduced plasma levels of active thymulin, the zinc-bound biologically active form (Zn-FTS) of the 'facteur timique serique' (FTS) (Dardenne et al, 1982). Peripheral low zinc bioavailability has been proposed as one of the mechanisms involved in cancer-related thymic immune deficiency (Fabris and Mocchegiani, 1995). Zinc is necessary for normal cell-mediated immunity (Chandra, 1985). However, the involvement of zinc deficiency in neoplasia remains unknown (Garofalo et al, 1979; Gorodetsky et al 1985; Fabris and Mocchegiani, 1995). Zinc is bound to various metalloproteins and other macromolecules produced by neoplastic cells (Bremner and May, 1989). Normal levels of zinc in neoplastic diseases are often associated with low peripheral zinc bioavailability (Fabris and Mocchegiani, 1995) because zinc tends to bind to proteins with a higher zinc binding affinity ($K_d$) than thymulin ($10^{-7}$ M) (Gastinel et al, 1984). In this context, $\alpha_2$-macroglobulin may play a key role for two reasons: firstly, because of a high $K_d$ for zinc ($10^{-10}$ M) (Giroux, 1975) and, secondly, because $\alpha_2$-macroglobulin is implicated as an inhibitor of matrix metalloproteases (MMPs) (Enghild et al, 1989) against invasive tumour proliferation (Stetler-Stevenson et al, 1993). Indeed, increases in $\alpha_2$-macroglobulin have been reported in experimental neoplasia (Mackiewicz et al, 1993) and in melanoma patients (Matoska et al, 1988). Therefore, we measured plasma levels of zinc, of active thymulin (Zn-FTS) and of $\alpha_2$-macroglobulin in patients affected by locally advanced cervical carcinoma. Because the non-zinc-bound form of thymulin (FTS) is inactive, and can be unmasked by in vitro zinc addition to plasma samples containing the total amount of thymulin (active Zn-FTS+inactive FTS) produced (Mocchegiani et al, 1994), we also tested total thymulin. NK activity and IL-2 plasma levels were measured, as well as plasma soluble IL-2 receptor (sIL-2R), because of its negative influence on IL-2 availability in solid tumours (Rubin et al, 1986; Pavlidis et al, 1995). In vitro zinc studies on IL-2 production from peripheral blood mononuclear cells (PBMCs) of cancer patients were carried out in view of a possible clinical trial with zinc.

*Received 14 October 1997*
*Revised 6 April 1998*
*Accepted 12 May 1998*

*Correspondence to:* E Mocchegiani, Immunology Centre, Research Department, INRCA, Via Birarelli 8, 60100, Ancona, Italy

### PATIENTS AND METHODS

### Patients

Blood samples were drawn from 22 patients with locally advanced squamous cervical carcinoma after radical surgery but with no previous treatment with antiblastic drugs. They were recruited from our series of cervical carcinoma patients at the Institute of Gynaecology and Obstetrics, Ancona University, Italy, from January 1994 to December 1995. Other inclusion criteria were: age ≤ 60 years; stage Ib2–IIb disease according to the FIGO classification (International Federation of Gynaecology and Obstetrics) (Grigsby 1996). Exclusion criteria were: present or past smoking; previous radiotherapy and/or chemotherapy; immunological diseases, including HIV seropositivity; immunosuppressive treatment in the last 4 months before the present study.

Twelve outpatients age matched and with the same socio-economic status, with normal cervical Pap smear and without immune or neoplastic diseases were used as healthy controls. All tests were performed with informed consent from patients and healthy control subjects.

### Thymulin determination

Plasma zinc-bound active thymulin (Zn-FTS), as extensively described elsewhere (Bach et al, 1975; Mocchegiani et al, 1994), was measured using a bioassay based on the ability to restore the inhibitory effect of azathioprine on rosette formation in spleen cells (RFCs) from young thymectomized mice. Results are expressed as $\log_{-2}$ of the reciprocal maximal dilution of tested plasma that induces this phenomenon (Bach et al, 1975). This technique is specific for thymulin, because the assay is unaffected by other thymic hormones, and the rosette-inducing activity is removed completely by passing plasma samples through an antithymulin immunoabsorbent (Bach et al, 1975). The sensitivity of this bioassay allows for the detection of 1 pg $ml^{-1}$ synthetic thymulin (Sigma, USA). Because in two consecutive blind assays no difference of more than one $\log_{-2}$ was found in all samples, the assay was considered reliable (Bach et al, 1975). This bioassay is still required because questions have been raised over the specificity of the radioimmunoassays (RIAs) developed up to now (Mocchegiani et al, 1994). To avoid possible interference due to zinc turnover, the thymulin bioassay was performed with zinc-enriched medium by in vitro addition of zinc sulphate at a final concentration of 200 nM, which has been shown to be the optimal concentration for unmasking inactive zinc-unbound thymulin (FTS) molecules and showing the total amount of thymulin produced (active Zn-FTS+inactive FTS) (Mocchegiani et al, 1994). The apparently low molar concentration of zinc required may be explained by the fact that the bioavailable free zinc is no more than 2–3% of total plasma zinc, the major quota being bound to proteins which are retained by membranes with a 50 000 molecular weight cut-off (Mocchegiani et al, 1994).

### Zinc and $\alpha_2$-macroglobulin plasma levels

Plasma zinc was measured using the method of Fernandez and Khan (1971). Blood samples were collected directly into fluorinated tubes (no. 115317, LP, Italy) in order to avoid possible contamination, and centrifuged 20 min later at 3000 ***g*** for 10 min. Plasma samples were frozen at –70°C and stored until used. Plasma zinc levels were determined by atomic absorption spectrophotometry (AAS) against zinc reference standards (Mocchegiani et al, 1994). The data were expressed in μg $dl^{-1}$.

Plasma $\alpha_2$-macroglobulin levels were tested using the nephelorimetric method (Boheringer, Italy). The data are expressed in mg $ml^{-1}$.

### IL-2 plasma determination

Interleukin 2 (IL-2) was measured in plasma by means of an enzyme-linked immunosorbent assay (Inter Test 2X Human IL-2 ELISA kit, Genzyme, USA) that is based on a sandwich principle using a monoclonal antibody in conjunction with polyvalent anti-IL-2 antibody. The results, expressed in pg $ml^{-1}$, are the average of two separate assays. The specificity of the determinations was confirmed in all cases by blocking interleukin 2 (by 60% or more) with a rabbit polyclonal antibody. The sensitivity of the Inter-test IL-2 kit ranged (95% confidence limits) between 100 and 2500 pg $ml^{-1}$.

### Soluble IL-2 receptor (sIL-2R) plasma determination

Plasma concentrations of sIL-2R were measured by means of an ELISA commercial kit (Predicta IL-2R, Genzyme that is based on the cleavage of IL-2R to yield sIL-2R using a biotinylated rabbit anti-IL-2R antibody conjugate with a peroxidase enzyme. The results, expressed in pg $ml^{-1}$, are the average of two separate assays and are referred to a standard curve. The sensitivity of the Predicta ELISA kit ranged (95% confidence limits) between 350 and 4000 pg $ml^{-1}$.

### NK cytotoxic assay

PBMCs were fractionated on Ficoll-Hipaque (Pharmacia, Sweden) and separated by density gradient centrifugation (400 ***g***, 30 min). Cells from the interface of the gradient were washed with phosphate-buffered saline (PBS) ($Ca^{2+}$ and $Mg^{2+}$ free), resuspended in RPMI-1640 (Gibco, Grand Island, NY, USA) plus 10% fetal calf serum (FCS, Gibco), and incubated in plastic Petri dishes for 1 h at 37°C in 5% carbon dioxide in air to remove adherent cells. The lymphocytes were then washed with RPMI-1640, counted and resuspended at a concentration of $2 \times 10^6$ $ml^{-1}$ in RPMI-1640 containing 10% decomplemented FCS, penicillin (100 U $ml^{-1}$) and streptomycin (100 μg $ml^{-1}$) (complete medium, CM). Viability was always greater than 98% as determined by trypan blue exclusion. The K562 tumour cell line, a myeloid cell line derived from a pleural effusion of a patient with a chronic myelocyte leukaemia in blast crisis, was used as the target cell. These cells were maintained in continuous culture throughout the study.

Briefly, NK cell assay was performed by a fluorimetric method, as extensively reported elsewhere (Provinciali et al, 1992), using a stock solution of carboxyfluorescein diacetate (c'FDA, Molecular Probes, OR, USA) (20 mg $ml^{-1}$ acetone, stored at –20°C) diluted in phosphate-buffered saline (PBS) to give a final concentration of 75 mg $ml^{-1}$. K562 target cells were used at a concentration of $1 \times 10^5$ $ml^{-1}$. Effector target cell ratios from 20:1 to 2.5:1 were tested in triplicate after 3 h incubation at 37°C in a humidifier under 5% carbon dioxide. The fluorescence in the supernatants was read with a Titertek Fluoroskan II (Flow Laboratories, USA). The percentage of specific lysis was calculated as follows:

$$\text{Percentage of specific lysis} = (F_{\text{med}} - F_{\text{exp}})/F_{\text{med}},$$

where $F$ represents the fluorescence of the solubilized cells after the supernatant has been removed, med is the fluorescence from target incubated in medium alone, and exp is the fluorescence

© Cancer Research Campaign 1999

from target incubated with effector cells. The data were expressed in lytic units (LU 20 per $10^6$ cells).

### In vitro cultures

Blood samples from adult healthy control subjects (five subjects) and cancer patients (five subjects) were diluted 1:2 with PBS ($Ca^{2+}$ and $Mg^{2+}$ free, Gibco), fractionated with Ficoll-Hipaque (Pharmacia, Sweden) and the PBMCs separated by density gradient centrifugation at 18°C for 30 min at 400 ***g***. The PBMCs were collected with a Pasteur pipette from the interface of the gradient, washed twice with PBS, counted and resuspended at a concentration of $1 \times 10^6$ cells $ml^{-1}$ in RPMI-1640 (Gibco, USA) containing penicillin (100 U $ml^{-1}$) and streptomycin (100 µg $ml^{-1}$) and then put in culture. The culture (in triplicate) of PBMCs without zinc or with zinc at the concentrations of 1 µM (physiological doses) (Dardenne et al, 1982; Mocchegiani et al, 1994) and 10 µM (up-physiological doses) (Dardenne et al, 1982; Mocchegiani et al, 1994) were incubated in zinc-free RPMI-1640 medium at 37°C in a humidifier under 5% carbon dioxide for 24 h. At the end of the incubation, the viability of cells was evaluated by means of trypan blue exclusion. The viability was greater than 98%. PBMCs were then centrifuged for 10 min at 400 ***g***. The supernatants obtained were harvested and IL-2 production was tested in the supernatants by means of Inter-test IL-2 ELISA kit (Genzyme). The data were expressed in pg $ml^{-1}$.

### Statistical analysis

The significance between the means was assessed by paired Student's *t*-test and Anova (one way) test. The correlations were determined by linear regression analysis by the least squares method. Differences were considered significant when $P < 0.05$.

### RESULTS

### Clinicopathological characteristics of cancer patients

The median age of our patients was 51.5 years (range 35–60 years). The median parity was 2 (range 1–4). Table 1 shows the clinicopathological characteristics of the patients. Eleven patients (50%) were in stage Ib2, four patients were in stage IIa and seven patients were in stage IIb of the neoplastic disease. Involvement of lymphnodes was observed in nine cases (41%) and lymphovascular invasion was present in 13 cases (59%). In addition, 17 cancer patients showed HPV DNA positivity. HPV DNA 16 is the most frequent type (71%) [polymerase chain reaction (PCR) technique].

**Table 1** Clinicopathological characteristics of cancer patients

| Parameters | No. cases | Percentage |
|---|---|---|
| FIGO stage | | |
| IB2 | 11 | 50 |
| IIA | 4 | 18 |
| IIB | 7 | 32 |
| Histological grade | | |
| G1 | 15 | 68 |
| G2 | 2 | 9 |
| G3 | 5 | 23 |
| Lymphovascular space invasion | | |
| | 9 | 41 |
| | 13 | 59 |
| Lymph node metastases | | |
| | 13 | 59 |
| | 9 | 41 |
| HPV DNA positivity | | |
| | 5 | 23 |
| | 17 | 77 |

### Plasma thymulin levels (active and total), plasma zinc concentrations and plasma $\alpha_2$-macroglobulin levels in cancer patients

Table 2 shows that active thymulin plasma levels (Zn-FTS) were reduced in cancer patients compared with age-matched healthy control subjects ($P < 0.003$). The total thymulin level (Zn-FTS + FTS) was not affected by the tumour, values being similar to those of healthy controls subjects (Table 2). Plasma zinc levels were in the normal range for age of the cancer patients (Table 2). However, some cancer patients, on an individual level, showed reduced zincaemia compared with healthy control subjects (data not shown).

Significant increases in $\alpha_2$-macroglobulin plasma concentrations are observed in cancer patients compared with healthy control subjects ($P < 0.001$) (Table 2).

When plasma zinc levels from healthy control subjects and cancer patients are plotted against the corresponding values of in vitro thymulin saturable fraction (ratio total thymulin ZnFTS+FTS/ active thymulin ZnFTS), a significant inverse correlation is found ($r = -0.60$; $P < 0.05$) (Figure 1). When plasma zinc levels or active thymulin concentrations from healthy control subjects and cancer patients were plotted against the corresponding values of plasma $\alpha_2$-macroglobulin, significant positive correlations were also observed ($r = 0.75$, $P < 0.01$; $r = 0.78$, $P < 0.01$, respectively).

**Table 2** Plasma active (Zn-FTS) and total (Zn-FTS+FTS) thymulin, zinc an $\alpha_2$-macroglobulin levels in cervical carcinoma patients

| | Healthy control subjects | Cervical carcinoma |
|---|---|---|
| Active thymulin (ZnFTS) ($log_{-2}$) | 3.0 ± 0.3 | 1.5 ± 0.5** |
| Total thymulin (ZnFTS+FTS) ($log_{-2}$) | 4.5 ± 0.3 | 4.5 ± 0.3 |
| Zinc (µg $dl^{-1}$) | 112.4 ± 16.1 | 100.7 ± 9.3 |
| $\alpha_2$-Macroglobulin (mg $dl^{-1}$) | 178.8 ± 17.2 | 260.0 ± 72.8* |

*$P < 0.003$ when compared with healthy age-matched control subjects. **$P < 0.001$ when compared with healthy age-matched control subjects.

 *© Cancer Research Campaign 1999*

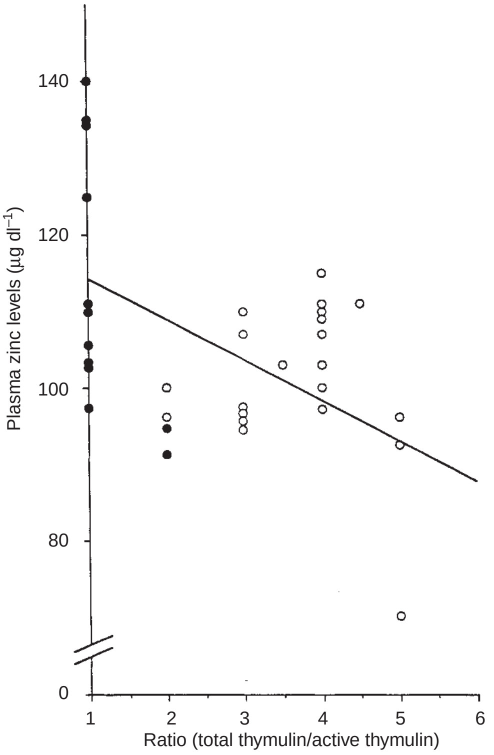

**Figure 1** Significant correlation ($r$ = 0.60, $P$ < 0.05) between plasma zinc levels and zinc-saturable thymulin fractions (total thymulin Zn-FTS+FTS/ active thymulin Zn-FTS) from the data of cancer patients (○) and of healthy age-matched control subjects (●)

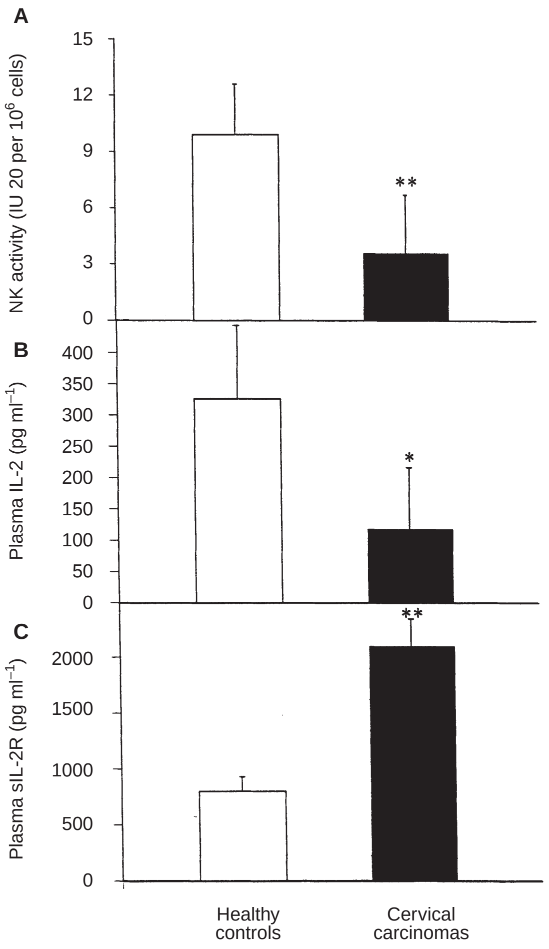

**Figure 2** NK activity (**A**), IL-2 (**B**) and sIL-2R (**C**) plasma levels in cancer patients (□) and in healthy age-matched control subjects (■) (means ± s.d.). *$P$ < 0.01 and **$P$ < 0.05 when compared with healthy control subjects

### Plasma IL-2 levels, plasma soluble IL-2 receptor (sIL-2R) concentrations and natural killer (NK) activity in cancer patients

Figure 2A and B shows that both NK activity and plasma IL-2 levels are reduced in cancer patients compared with healthy control subjects ($P < 0.05$ and $P < 0.01$ respectively). Concomitantly, significant increases of plasma sIL-2R concentrations were observed in cancer patients compared with healthy controls ($P < 0.01$) (Figure 2C). When the data for IL-2 or sIL-2R from healthy control subjects and cancer patients were plotted against the corresponding values of NK activity, significant positive or negative correlations were found for IL-2 or sIL-2R ($r = 0.78$, $P < 0.01$; $r = -0.71$, $P < 0.01$ respectively).

When the data for zinc and IL-2 from healthy control subjects and cancer patients were plotted against the corresponding values for NK activity, significant correlations were observed ($r = 0.80$, $P < 0.01$; $r = 0.78$, $P < 0.01$, respectively), as well as between NK activity and active thymulin or thymulin saturable fraction ($r = 0.92$, $P < 0.01$; $r = 0.90$, $P < 0.01$, respectively).

### Effect of in vitro zinc addition on IL-2 production in the supernatants from PBMCs of cancer patients

Table 3 shows IL-2 production in the supernatants from PBMCs of cancer patients and healthy control subjects after in vitro zinc addition [physiological (1 μM) or up-physiological (10 μM) dose]. Significant increases in IL-2 production are observed in the supernatants from PBMCs of cancer patients after in vitro zinc addition at a concentration of 1 μM compared with values without zinc ($P < 0.001$). However, it is noteworthy that more significant increases in IL-2 production are present when zinc is added at 10 μM compared with zinc at 1 μM (79.9 pg ml$^{-1}$ vs. 50.6 pg ml$^{-1}$ of IL-2) (Table 3). When zinc (1 μM) was added to cultures of PBMCs from healthy control subjects, no substantial modifications of IL-2 production were observed compared with PBMC cultures without zinc (Table 3). IL-2 production was decreased in healthy controls after in vitro zinc addition at 10 μM compared with those controls without zinc or with zinc 1 μM ($P < 0.01$) (Table 3).

### DISCUSSION

Active thymulin (Zn-FTS) was reduced in patients affected by locally advanced cervical carcinoma, whereas total thymulin level (active thymulin Zn-FTS+inactive thymulin FTS) was in the normal range. Plasma zinc levels were within the normal range for the age of the cancer patients, whereas $\alpha_2$-macroglobulin was increased. NK activity as well as IL-2 production were reduced. Concomitantly, sIL-2R was increased.

Cell-mediated immunity is decreased in neoplasia (Burnet, 1971; Broder and Negson, 1982). The causes are still unclear and undefined (Dilman, 1977). We suggest that tumour-associated

**Table 3** Effect of in vitro zinc addition on IL-2 production from PBMCs of cancer patients

| | Dose of zinc | IL-2 production (pg ml$^{-1}$) |
|---|---|---|
| Cervical carcinoma | Without zinc | 29.3 ± 8.7 |
| | Zinc 1 μM | 50.6 ± 9.2* |
| | Zinc 10 μM | 79.9 ± 8.5* |
| Healthy control subjects | Without Zinc | 178.4 ± 6.5 |
| | Zinc 1 μM | 178.4 ± 6.6 |
| | Zinc 10 μM | 123.5 ± 8.5** |

*$P < 0.001$ when compared with the values without zinc. **$P < 0.01$ when compared with the values of healthy controls without zinc or zinc 1 μM.

immunodeficiency may depend on changes in thymic endocrine activity, which is necessary for good cell-mediated immunity (Goldstein, 1984).

Although histological evidence of thymic abnormalities in neoplasia is not currently available (Hamoudi et al, 1982), the level of one of the best-known thymic hormones, i.e. thymulin, has been found to be reduced in cancer (Mocchegiani et al, 1990, 1994). In agreement with these studies, a significant reduction in active thymulin has also been observed in patients with locally advanced cervical carcinoma. Low peripheral bioavailability of zinc, which plays a pivotal role in normal immune function (Chandra, 1985), may be involved. Indeed, zinc is required for the biological activity of thymulin (Zn-FTS) (Dardenne et al, 1982), whereas the non zinc-bound form (FTS) is inactive with an inhibitory action on the active form (Zn-FTS) (Mocchegiani et al, 1994) probably competing with FTS receptors as do FTS analogues (Pleau et al, 1979). In vitro addition of zinc to plasma samples containing FTS is able to unmask the inactive form (FTS), revealing the total amount of thymulin (active+inactive) in the circulation (Mocchegiani et al, 1994). The ratio of total thymulin to active thymulin is the thymulin fraction that is saturable by zinc ions and represents a good marker of true zinc deficiency and, consequently, of peripheral zinc which may be low in spite of plasma zinc levels in the normal range (Fabris and Mocchegiani, 1995). This thymulin methodological procedure performed in solid tumours and in leukamia has allowed us to demonstrate that reduced plasma levels of active thymulin (Zn-FTs) are largely due to the presence of non-zinc-bound inactive thymulin molecules (FTS) not saturated by zinc ions despite zinc levels in the normal range (Mocchegiani et al, 1990; Mocchegiani et al 1994). Supplementary zinc restores the thymic endocrine defect (Fabris and Mocchegiani, 1995).

Because atomic absorption spectrophotometry (AAS) measures free and patients with bound zinc (Fernandes and Khan, 1971), normal plasma zinc levels for the age are observed in patients with cervical carcinoma. This fact is misleading because, when plasma zinc is plotted against the saturable thymulin fraction, a significant inverse correlation is found (Figure 1), suggesting low peripheral zinc bioavailability results in saturation of non-zinc-bound thymulin molecules produced in this type of cancer. Such an assumption is supported because the fact that total thymulin does not differ in cancer patients and healthy control subjects.

Such a phenomenon may occur because zinc is required for the biological activity of many enzymes and metalloproteins, which bind zinc with different binding affinities ($K_d$) (ranging from $10^{-10}$ M for $\alpha_2$-macroglobulin to $10^{-3}$ M for angiotensin-converting enzyme) (Bremner and May, 1989). Thus, by the low peripheral zinc bioavailability might occur because of increased concentrations of some zinc-binding proteins. In this context, $\alpha_2$-macroglobulin may play a crucial role for two reasons: first its binding affinity ($K_d$ for zinc ($10^{-10}$ M) (Giroux, 1975), higher than that of thymulin ($10^{-7}$ M) (Gastinel et al, 1984); secondly, $\alpha_2$-macroglobulin is an inhibitor of matrix metalloproteases (MMPs), which are involved in tumour cell proliferation (Stetler-Stevenson et al, 1993); a direct interaction between MMPs and $\alpha_2$-macroglobulin removes proteolytic potential from the circulation (Enghild et al, 1989). This direct inhibitory role has been considered to be a reserve when other inhibitors [tissue inhibitors of matrix metalloproteinases (TIMPs)] are deficient (Chu et al, 1994). MMPs are induced temporarily in response to exogenous signals such as various cytokines produced by macrophages, mainly IL-1, IL-6 and TNF (Kahari and Saarialho Kere, 1997), which are increased in cancer (Dinarello, 1984). $\alpha_2$-macroglobulin binds these cytokines (Chu et al, 1994) in order to induce latency on cytokines (James, 1990). In vitro studies have shown that zinc is required for this binding (Getting and Crews, 1994). $\alpha_2$-macroglobulin thus exerts this indirect inhibitory action on MMPs by means of the cytokine-binding inducing latency on cytokines themselves, and consequently, minor MMPs production. This may be one of the possible inhibitory mechanisms of $\alpha_2$-macroglobulin against MMPs, as suggested by James (1990) and Chu et al (1994). Thus, increased $\alpha_2$-macroglobulin levels are expected in cervical carcinoma not as an epiphenomenon but as a cause or a consequence of the tumour and, as such, a necessary protective biological event. Moreover, this protective role of $\alpha_2$-macroglobulin may further justify the shift of zinc towards this metalloprotein in cervical carcinoma. On the other hand, increases in $\alpha_2$-macroglobulin have been also found in tumour experimental models (Mackiewicz et al, 1993) and in melanoma patients, although such increments were associated with unfavourable prognosis (Matoska et al, 1988). This is not surprising because the shift of zinc results in a low zinc bioavailability with consequent reduction in peripheral thymulin molecules saturated by zinc ions, resulting in reduced zinc-bound active thymulin and impairment of immune efficiency against tumours. The existence of significant correlations between zinc or active thymulin and $\alpha_2$-macroglobulin in cancer patients may be in line with this interpretation; while positive correlation between plasma zinc and active thymulin (ZnFTS) is obvious because many thymulin molecules are present in zinc-unbound form (FTS) due to zinc sequestration by $\alpha_2$-macroglobulin with consequent increments of this metalloprotein in cancer patients (see Table II). Such a phenomenon occurs for metallothioneins in leukaemia characterized by normal zincaemia and reduced levels of active thymulin (Mocchegiani et al, 1994). Despite metallothioneins having been suggested as donors of zinc for thymulin activation in thymic epithelial cells (TEC) (Savino et al, 1984) such a donation does not occur in leukemia, and more in general in cancer, because of the reactivation of thymulin after in vitro zinc addition up to plasma samples (Mocchegiani et al, 1994). Indeed supplementing zinc corrects both thymic and peripheral immune defects in cancer (Fabris and Mocchegiani, 1995). Thus, taking into account the role of thymulin (Goldstein, 1984) and zinc (Chandra, 1985) for cell-mediated immunity, the reduced active thymulin levels may be relevant for the efficiency of peripheral immune functions in cervical carcinoma, because the major quota of zinc bound with $\alpha_2$-macroglobulin. Thereby, while one hand increased $\alpha_2$-macroglobulin in cancer may be useful as

 © Cancer Research Campaign 1999

protective agent, on the other hand it may be dangerous for immune efficiency, as recently also suggested for increased metallothioneins in ageing: from protective in young age to dangerous role in old age for immune functions (Mocchegiani et al, 1997).

Experimental evidence shows that zinc-bound active thymulin (ZnFTS) affects both in vivo (Bardos and Bach, 1982) and in vitro NK activity (Muzzioli et al, 1992), which is, in turn, under the control of IL-2 (Henney et al, 1981). In addition, zinc affects IL-2 activity (Tanaka et al, 1990). Natural Killer (NK) activity and cytotoxic-T cell response (CTLs) are important to test the cytotoxic functions (Bontkes et al, 1997). However a minority of CTLs are activated in cervical carcinoma and the cytotoxic activity is very low and so not explicative to really document cytotoxic function in cervical carcinoma (Park and Kim, 1989; Bontkes et al 1997). The Natural Killer activity seems a more reliable cytotoxic test (Park and Kim, 1989; Bontkes et al, 1997).

Indeed, the relevance of NK activity to the clinical outcome of patients with cervical carcinoma has been well documented (Garzetti et al, 1995). In addition, decreased IL-2 production as a risk factor for the progression of cervical carcinoma (Shiffman and Brinton, 1995) has also been shown. In agreement with these studies, NK activity and IL-2 production are both decreased in cervical carcinoma. Such decreases are correlated with increases in soluble IL-2 receptor (sIL-2R) in the circulation. Because sIL-2R has a negative influence on the IL-2 availability in solid tumours (Rubin et al, 1986; Pavlidis et al, 1995), this might explain the reduced plasma IL-2 levels found in cancer patients, and it gives further support to the importance of IL-2 and NK activity in cervical carcinoma. Reduction in NK activity, low bioavailability of IL-2 and increased circulating sIL-2R are present in some types of cancer (Gooding et al, 1995; Wasik et al, 1996), characterized by slight zinc deficiency (Fabris and Mocchegiani, 1995).

Thus, reduced IL-2, together with the knowledge that active thymulin is also involved in IL-2 production (Goldstein, 1984) and on NK activity (Bardos et al, 1982; Muzzioli et al, 1992), suggests the crucial role played by zinc and thymulin for IL-2 and NK activity in cervical carcinoma. The presence of significant correlations between plasma zinc levels and plasma IL-2 concentrations and between NK activity and plasma zinc levels or active thymulin concentrations or the zinc-saturable thymulin fraction may support this interpretation.

Intriguing points are also related to the role of zinc in the extrathymic T-cell pathway (Mocchegiani et al, 1997), which is important for NK-cell maturation in tumours (Seki et al, 1991). Thus, good zinc bioavailability may be crucial for the effectiveness of NK cells of thymic and extrathymic origin during cancer development. Zinc is also required by macrophages for TNF-$\alpha$ production (Driessen et al, 1994), which is, in turn, involved either in NK activity (Ogata et al, 1995) or in apoptosis in tumour cell lines through its transcriptional factor NF-$\kappa$B (VanAntwerp et al, 1996). These findings, together with our data, suggest a possible beneficial effect of zinc treatment on the efficiency of the entire immune system in cervical carcinoma, as documented in other types of cancer (Fabris and Mocchegiani, 1995).

However, the recent discovery that zinc induces mitogenesis (Cunningham-Rundles et al, 1990) must not be discounted. However, recent "in vitro" studies found no mitogenic role of zinc (physiological dose) on lymphoblastoid cells (Mocchegiani et al, 1994).

Interestingly, "in vitro" zinc (physiological or up-physiological dose) induces increases in IL-2 production in the supernatants from PBMCs of cancer patients, despite the fact that intracellular zinc content of PBMCs is not different between cancer patients and healthy control subjects (E. Mocchegiani, unpublished observation). This suggests that exogenous zinc is necessary for good IL-2 production by lymphocytes of cancer patients, as suggested previously for aging people (Tanaka et al, 1990). "In vitro" zinc (0.25 mM) induces a decrease in IL-2 production from PBMCs of adult healthy humans (Driessen et al, 1994). In agreement with this last study, we observed a decrease in IL-2 production from PBMCs of healthy control subjects at zinc concentrations of 10 $\mu$M. These findings may confirm a toxic role for zinc in immune functions at up-physiological or pharmacological doses in healthy subjects (Chandra, 1984), but, in contrast they suggest the importance of low zinc bioavailability in impaired immune efficiency in cervical carcinoma.

The mechanism(s) by which "in vitro" zinc affects immune cell production of IL-2 could be a direct effect on T-cells by means of a receptor–MHC complex (Miller and Strittmatter, 1992) or by means of membrane protein kinase C (PKC) (Csermely et al, 1988). Alternatively, indirect mechanisms mediated macrophage-produced IL-12, which, in turn, induces Th 1 cells to produce IL-2 (Trinchieri and Scott 1994) may be involved. In any case, zinc increases IL-2 production from PBMCs of cancer patients, providing a further rationale for physiological zinc treatment in cervical carcinoma.

In conclusion, the central (thymic endocrine activity) and peripheral immune defects (NK and IL-2) might be due to increased $\alpha_2$-macroglobulin in cervical carcinoma. Whether the defect of NK activity is due to a low free quota of zinc or to reduced thymulin activity remains to be clearly established.

### ACKNOWLEDGEMENTS

This paper was supported by INRCA, by Italian Health Ministry and by the Institute of Gynaecology and Obstetrics, University of Ancona, Italy. The authors are indebted to Mrs Nazzarena Gasparini, Miss, Rosalia Stecconi and Mr Flavio Marchegiani for their excellent technical assistance. We also thank Mr Graziano Mazzarini and Mr Gianni Bernardini for graphic design and Mrs Monica Glebocki for reading the text.

### REFERENCES

Bach JF, Dardenne M, Pleau JM and Bach MA (1975) Isolation, biochemical characteristics and biological activity of a circulating thymic hormone in the mouse and in the human. *Ann NY Acad Sci USA* **249**: 186–193

Bardos P and Bach JF (1982) Modulation of mouse natural killer cell activity by the serum thymic factor. *Scand J Immunol* **12**: 321–325

Bontkes HJ, de Gruijl TD, Walboomers JM, van den Muysemberg AJ, Gunther AW, Scheper RJ, Mejier CJ and Kummer JA (1997) Assessment of cytotoxic T-lymphocyte phenotype using the specific markers granzyme B and TIA-1 in cervical neoplastic lesions. *Br J Cancer* **76**: 1353–1360

Bremner I and May PM (1989) Systemic interaction of zinc. In *Zinc in Human Biology*. Mills CF (ed.), pp. 95–108. Springer-Verlag: London

Broder S and Negson M (1982) The interrelationship between cancer and immune deficiency. In *Cancer in the Young*. Levin AS (ed.), pp. 29–41. Publishing USA: New York

Burnet FM (1971) Immunological surveillance in neoplasia. *Transplant Rev* **7**: 3–13

Chandra RK (1984) Excessive intake of zinc impairs immune response. *J Am Med Assoc* **252**: 1443–1446

Chandra RK (1985) Trace elements regulation and immunity and infection. *J Am Coll Nutr* **4**: 5–16

Chu CT, Howard GC, Misra UK and Pizzo SV (1994) α-2 macroglobulin: a sensor of proteolysis. *Ann NY Acad Sci USA* **737**: 291–307

Csermely P, Szarme M, Resch K and Somogy J (1988) Zinc can increase the activity of protein kinase C and contributes to its binding to plasma membranes in T lymphocytes. *J Biochem Chem* **263**: 6487–6490

Cunningham-Rundles S, Bockman RS, Lin A, Giardina PV, Hilgartner MW, Caldwell-Brown D and Carter DM (1990) Physiological and pharmacological effects of zinc on immune response. *Ann NY Acad Sci USA* **587**: 113–118

Dardenne M, Pleau JM, Nabama B, Lefancier P, Denien M, Choay J and Bach JF (1982) Contribution of zinc and other metals to the biological activity of the serum thymic factor. *Proc Natl Acad Sci USA* **79**: 5370–5373

Dilman VM (1977) Metabolic immunodepression which increases the risk of cancer. *Lancet* **ii**: 1207–1210

Dinarello CA (1984) Interleukin 1. *Rev Infect Dis* **6**: 51–94

Driessen C, Hirv K and Kirchner H (1994) Induction of cytokines by zinc ions in human peripheral blood mononuclear cells and separated monocytes. *Lymphokines Cytokines Res* **13**: 15–20

Enghlid JJ, Salvesen G, Brew K and Nagase H (1989) Interaction of human rheumatoid synovial collagenase (matrix metalloproteinase 1) and stromelysin (metalloproteinase 3) with human α-2 macroglobulin and chicken ovostatin. Binding kinetics and identification of matrix metalloproteinase cleavage sites. *J Biol Chem* **264**: 8779–8785

Fabris N and Mocchegiani E (1995) Zinc, human diseases and aging. A review. *Aging Clin Exp Res* **7**: 77–93

Fernandez FJ and Khan H (1971) Clinical methods for atomic absorption spectroscopy. *Clin Chem News Lett* **3**: 24–28

Garofalo JA, Strong E, Cunningham-Rundles S, Erlandson E, Mendez-Botet C, Schawartz M and Good RA (1979) Serum zinc in patients with epidermial cancer of the head and neck. *Fed Proc* **38**: 713–718

Garzetti GG, Ciavattini A, Lucarini G, Goteri G, De Nictolis M and Biagini G (1996) Microinvasive cervical carcinoma and cervical intraepithelial neoplasia: biologic significance and clinical implications of 72-kDa metalloproteinase immunostaining. *Gynecol Oncol* **61**: 197–203

Gastinel LN, Dardenne M, Pleau JM and Bach JF (1984) Studies on the zinc binding site to the serum hymic factor. *Biochim Biophys Acta* **797**: 147–155

Getting PGW and Crews BC (1994) Binding of epidermal growth factor to human α-2 macroglobulin. Significance for cytokines α-2 macroglobulin interactions. *Ann NY Acad Sci USA* **737**: 383–398

Giroux EL (1975) Determination of zinc distribution between albumin and α-2 macroglobulin in human serum. *Biochem Med* **12**: 258–266

Goldstein AL (1984) *Thymic Hormones and Lymphokines*. Plenum Press: New York

Gooding R, Riches P, Dadian G and Gore M (1995) Increased soluble interleukin-2 receptor concentration in plasma predicts a decreased cellular response to IL-2. *Br J Cancer* **72**: 452–455

Gorodetsky R, Fuks Z, Sulkes A, Ginsburg H and Wesheler Z (1985) Correlation of erythrocyte and plasma levels of zinc, copper and iron with evidence of metastatic spread in cancer patients. *Cancer* **55**: 779–786

Grigsby PW (1996) Stage IB1 vs IB2 carcinoma of the cervix: should the new FIGO staging system define therapy. *Gynaecol Oncol* **62**: 135–136

Hamoudi AB, Newton Jr WA, Mancer R and Penn GD (1982) Thymic changes in histiocitosis. *Am J Clin Pathol* **77**: 169–172

Henney CS, Kuribayashi K, Kern DE and Gills S (1981) Interleukin-2 augments natural killer cell activity. *Nature* **291**: 335–340

Kahari VM and Saarialho-Kere U (1997) Matrix metalloteinase in skin. A review. *Exp Dermatol* **6**: 199–213

James K (1990) Interactions between cytokines and α-2 macroglobulin. *Immunol Today* **11**: 163–166

Mackiewicz A, Kushner I and Baumann H (1993) *Acute Phase Proteins: Molecular Biology, Biochemistry, Clinical Application*. CRC Press: Boca Raton, FL

Matoska J, Wahlstrom T, Vaheri A, Bizik J and Grofova M (1988) Tumour associated α-2 macroglobulin in human melanoma. *Int J Cancer* **41**: 359–363

Miller GG and Strittmatter WJ (1992) Identification of human T-cells that require zinc for the growth. *Scand J Immunol* **36**: 269–277

Mocchegiani E, Cacciatore L, Talarico M, Lingetti M and Fabris N (1990) Recovery of low thymic hormone levels in cancer patients by lysine–arginine combination. *Int J Immunopharmacol* **12**: 365–371

Mocchegiani E, Paolucci P, Granchi D, Cavallazzi L, Santarelli L and Fabris N (1994) Plasma zinc level and thymic hormone activity in young cancer patients. *Blood* **83**: 749–757

Mocchegiani E, Verbanac D, Santarelli L, Tibaldi A, Muzzioli M, Radosevic-Stasic B and Milin C (1997) Zinc and metallothioneins on cellular immune effectiveness during liver regeneration in young and old mice. *Life Sci* **61**: 1125–1145

Muzzioli M, Mocchegiani E, Bressani N, Bevilacqua P and Fabris N (1992) In vitro restoration by thymulin of NK activity of cells from old mice. *Int J Immunopharmacol* **14**: 57–61

Ogata K, Tamura H, Yokose N, An E, Dan K, Hamaguchi H, Sakamaki H, Onozawa Y, Clark SC and Nomura T (1995) Effects of interleukin 12 on natural killer cell cytotoxicity and the production of interferon gamma and tumour necrosis factor-alfa in patients with myelodysplastic syndromes. *J Haematol* **90**: 15–21

Park TK and Kim SN (1989) Cell-mediated immunity in patients with invasive carcinoma of the cervix. *Yonsei Med J* **30**: 164–172

Pavlidis NA, Bairaktari E, Kalef-Ezra J, Nicolaides C, Seferiadis C and Fountzilas G (1995) Serum soluble interleukin-2 receptors in epithelial ovarian cancer patients. *Int J Biol Markers* **10**: 75–80

Pleau JM, Dardenne M, Blanot B, Bricas E and Bach JF (1979) Antagonist analogues of serum thymic factor FTS interacting with the FTS cellular receptor. *Immunol Lett* **1**: 179–184

Provinciali M, Di Stefano G and Fabris N (1992) Optimization of cytotoxic assay by target cell retention of the fluorescent dye carboxyfluorescin diacetate (CFDA) and comparison with conventional $^{51}$Cr release assay. *J Immunol Methods* **155**: 9–24

Rani S, Vaidya MC and Rani K (1992) Role of cell growth factor (interleukin-2) and its receptors in carcinoma cervix patients. *J Steroid Biochem Mol Biol* **41**: 837–839

Robertson MJ and Ritz J (1990) Biology and clinical relevance of human natural killer cells. *Blood* **12**: 2421–2438

Rubin LA, Jay G and Nelsen DL (1986) The released interleukin-2 receptors bind interleukin-2 efficiently. *J Immunol* **137**: 3841–3844

Savino W, Huang PC, Corrigan A, Berrih S and Dardenne M (1984) Thymic hormone-containing cells. V. Immunohistological detection of metallothionein within the cells bearing thymulin (a zinc-containing hormone) in human and mouse thymuses. *J Histochem Cytochem* **32**: 942–946

Seki S, Abo T, Sugiura K, Otheki T, Kobata T, Yagita H, Okumura K, Rikiishi H, Masuda T and Kumagai K (1991) Reciprocal T-cell response in the liver and in the thymus of mice injected with syngenic tumour cells. *Cell Immunol* **137**: 46–54

Shiffman MH and Brinton LA (1995) The epidemiology of cervical carcinogenesis. *Cancer* **76**: 1888–1901

Stetler-Stevenson WG, Liotta LA and Kleiner Jr DE (1993) Extracellular matrix 6: role of matrix metalloproteinase in tumour invasion and metastasis. *FASEB J* **7**: 1434–1441

Tanaka Y, Shiozawa S, Moromoto I and Fujita T (1990) Role of zinc in interleukin-2 (IL-2)-mediated T-cell activation. *Scand J Immunol* **31**: 547–552

Trinchieri G and Scott P (1994) The role of interleukin 12 in the immune response, diseases and therapy. *Immunol Today* **15**: 460–463

Van Antwerp DJ, Martin SJ, Kafri T, Green DR and Verma IM (1996) Suppression of TNF-alfa-induced apoptosis by NF-kB. *Science* **274**: 787–789

Wasik K, Vonderheid EC, Bigler RD, Marti R, Lessin SR, Polanski M and Kadin ME (1996) Increased serum concentration of the soluble interleukin-2 soluble receptor in cutaneous T-cell lymphoma. *Arch Dermatol* **132**: 42–47

 *© Cancer Research Campaign 1999*
